# Quantifications of CSF Apoptotic Bodies Do Not Provide Clinical Value in Multiple Sclerosis

**DOI:** 10.3389/fneur.2019.01241

**Published:** 2019-11-26

**Authors:** Ruturaj Masvekar, Jordan Mizrahi, John Park, Peter R. Williamson, Bibiana Bielekova

**Affiliations:** National Institute of Allergy and Infectious Diseases, National Institutes of Health, Bethesda, MD, United States

**Keywords:** multiple sclerosis, cerebrospinal fluid, apoptotic bodies, clinical outcomes, flow cytometry, cell surface markers

## Abstract

Multiple sclerosis (MS) is an inflammatory disease of the central nervous system (CNS) that leads to the death of neurons and oligodendrocytes, which cannot be measured in living subjects. Physiological cellular death, otherwise known as apoptosis, progresses through a series of stages which culminates in the discharge of cellular contents into vesicles known as apoptotic bodies (ABs) or apoptosomes. These ABs can be detected in bodily fluids as Annexin-V-positive vesicles of 0.5–4.0 μm in size. In addition, the origin of these ABs might be detected by staining for cell-specific surface markers. Thus, we investigated whether quantifications of the total and CNS cell-specific ABs in the cerebrospinal fluid (CSF) of patients provided any clinical value in MS. Extracellular vesicles, from CSF of 64 prospectively-acquired subjects, were collected in a blinded fashion using ultra-centrifugation. ABs were detected by flow cytometry using bead-enabled size-gating and Annexin-V-staining. The origin of these ABs was further classified by staining the vesicles for cell-specific surface markers. Upon unblinding, we evaluated the differences between diagnostic categories and correlations with clinical measures. There were no statistically significant differences in the numbers of total or any cell-specific ABs across different disease diagnostic subgroups and no significant correlations with any of the tested clinical measures of CNS tissue destruction, disability, MS activity, and severity (i.e., rates of disability accumulation). Overlap of cell surface markers suggests inability to reliably determine origin of ABs using antibody-based flow cytometry. These negative data suggest that CNS cells in MS either die by non-apoptotic mechanisms or die in frequencies indistinguishable by current assays from apoptosis of other cells, such as immune cells performing immunosurveillance in healthy conditions.

## Introduction

Multiple sclerosis (MS) is a chronic immune-mediated disease of the central nervous system (CNS), leading to the demyelination of axons and neurodegeneration. Alongside traumatic brain injury, it is the most frequent cause of neurological disability in young adults (1). Findings from prior studies led to the hypothesis that MS can be largely divided into two stages, starting with the inflammatory phase in the periphery and later entering into the neurodegenerative phase (2). Although great progress has been made in understanding the inflammatory components of the disease, the neurodegenerative components are still obscure.

Currently, there are two main ways to measure neurodegenerative process in living human subjects: structural imaging and measurement of neurofilament light chain protein (NFL) (3, 4). Structural (MRI) imaging identifies CNS tissue destruction as brain/spinal cord atrophy. However, MRI imaging fails to provide cellular or molecular information and therefore cannot reliably measure the loss of crucial cell types such as neurons and oligodendrocytes, especially when loss of these CNS cells may be masked by infiltration of CNS tissue by immune cells or by compensatory astrogliosis. With emergence of ultra-sensitive Single Molecule Analysis (Simoa) NFL assay (5, 6) blood (serum or plasma) NFL levels can be measured both in healthy subjects and MS patients. In MS NFL levels are increased during MS activity and they can weakly predict subsequent MS progression on a group, but not individual levels (4, 7–11). Thus, there is still a need to develop biomarkers of neuronal and oligodendroglial injury/loss that can be applied on a patient level.

Pathology studies have shown the presence of apoptotic cells, mainly oligodendrocytes and neurons, at the site of MS lesions (2, 12, 13), suggesting that CNS cell apoptosis plays an important role in the irreversible neurological disability during the progressive stage of MS. This hypothesis was further supported by animal modeling with antiapoptotic protein B-cell lymphoma-2 (Bcl-2)-overexpressing transgenic mice; in comparison with wild type (WT) these transgenic mice showed reduced experimental autoimmune encephalomyelitis (EAE)-severity, despite similar inflammatory response (14).

Apoptotic cells progress through a series of stages including chromatin condensation, DNA fragmentation, membrane blebbing, and cell shrinkage, which all culminate in the discharge of cellular contents into extracellular vesicles, known as apoptotic bodies (ABs) or apoptosomes (15, 16). Previous studies have tried to isolate and identify ABs from subject's body fluid and use them as markers of respective disease-related degenerative processes (17–20).

Thus, the goal of the current study was to identify the presence of apoptotic cells in the CNS of living subjects by measuring the total and cell-specific ABs in cerebrospinal fluid (CSF) of patients. Additionally, we asked whether densities of total or cell-specific ABs differentiate MS from healthy donors (HDs), and within MS patient cohorts correlate with clinical measures of CNS tissue destruction, disability, MS activity and severity (i.e., rates of disability accumulation).

## Materials and Methods

### Cell Cultures and Treatments

Human neuroblastoma cells (SK-N-SH; ATCC# HTB-11, Manassas, VA) were cultured on poly-L-lysine (PLL; Sigma-Aldrich, St. Louis, MO) -coated plates (Costar, Corning, NY), in Dulbecco's modified Eagles medium (DMEM; Gibco, Gaithersburg, MD) supplemented with fetal bovine serum (FBS; Gemini Bio-Products, Sacramento, CA), and sodium pyruvate (Lonza, Walkersville, MD). Cells were either left untreated (Control) or treated with Staurosporine (0.5 μM; R&D Systems Inc., Minneapolis, MN). Twenty four hours after treatment, culture supernatants were collected and stored on ice until further use. Cells were washed with phosphate-buffered saline (PBS; Gibco, Gaithersburg, MD) and detached from plate using trypsin-EDTA solution (Sigma-Aldrich); detached cells were pelleted and stored on ice until further use.

### CSF Collection and Processing

All subjects were recruited under IRB-approved protocols (Comprehensive Multimodal Analysis of Neuroimmunological Diseases of the Central Nervous System, ClinicalTrials.gov Identifier: NCT00794352; and Evaluation and Follow-up of Patients with Cryptococcosis, ClinicalTrials.gov Identifier: NCT00001352) and all patients provided written informed consent. CSF from subjects were collected per standardized operating procedures (21). CSF aliquots were prospectively labeled using alphanumeric code, stored on ice until further use and analyzed in a blinded fashion.

### Isolation of Apoptotic Bodies

ABs were isolated from culture supernatants and CSF as previously described (19, 22–24). Briefly, cells were isolated and removed by pelleting at 335 g for 10 min. To remove cell-debris, cell-free supernatants were centrifuged at 1,000 g for 10 min; followed by another centrifugation at 2,000 g for 30 min to pellet ABs. Pelleted ABs were resuspended and washed with PBS.

### Flow Cytometry

ABs were stained with Annexin V-FITC, and cells were stained with Annexin V-FITC and Propidium Iodide (TACS® Annexin V Kit; Trevigen Inc., Gaithersburg, MD) as per manufacturer's instructions. CSF ABs were also stained for CNS cell-specific surface markers to identify their origin: We used cell-surface markers previously employed in isolation of human CNS cells from brain specimens using immune-panning, and validated by cell-specific RNA profiles (25, 26): CD90 (Neuronal surface marker; Human CD90/Thy1 APC-conjugated Antibody; R&D Systems, Minneapolis, MN; Clone # Thy-1A1), HepaCAM (Astroglial surface marker; Human HepaCAM Antibody; R&D Systems; Clone # 419305; tagged with DyLight 405; Novus Biologicals, Centennial, CO), GalC (Oligodendroglial surface marker; Anti-Galactocerebroside Antibody; EMD Millipore, Burlington, MA; Clone # mGalC; tagged with PerCP-Cy5.5; Novus Biologicals), CD31 (Endothelial cell surface marker (27); Human CD31/PECAM-1 PE-conjugated Antibody; R&D Systems; Clone # 9G11), and CD14 (Myeloid lineage cell surface marker (28); Alexa Fluor® 700 anti-human CD14 Antibody; BioLegend, San Diego, CA; Clone # HCD14). Briefly, after wash with PBS, pelleted ABs were resuspended in Annexin V-FITC and fluorescence-tagged antibodies against cell-specific surface markers in Annexin V-binding buffer (provided with TACS® Annexin V Kit) and incubated in dark for 15 min at room temperature. Stained ABs were washed with binding buffer and then analyzed using fluorescence-activated flow cytometer (BD LSR II Flow Cytometer, BD Biosciences, San Jose, CA). Gating on ABs included size gate [1–4 μm (29)], using Flow Cytometry Size Calibration Kit (ThermoFisher Scientific, Grand Island, NY). The vesicles in 1–4 μm size gate were further analyzed for Annexin V and cell-specific surface markers' staining.

### Subjects' Demographics Data

A total of 64 CSF samples were analyzed. After unblinding diagnostic codes, this cohort consisted of healthy donors (HD, *n* = 10), non-inflammatory neurological disorders (NIND, *n* = 5), other inflammatory neurological disorders (OIND, *n* = 12; mainly, comprised of Cryptococcal Meningitis patients), clinically isolated syndrome that did not yet fulfill MS diagnostic criteria (CIS, *n* = 2), relapsing-remitting MS (RR-MS, *n* = 17), and progressive MS [P-MS, comprised of both secondary- (SP-MS) and primary-progressive MS (PP-MS), *n* = 18] (Table 1). MS diagnostic subgroups (CIS, RR-MS, SP-MS, and PP-MS) were classified using McDonald's criteria, 2010 revisions (30). MS cohort (both RR- and P-MS) was further separated based on disease activity (active vs. non-active MS) using clinical relapses and new contrast-enhancing or new MRI lesions.

**Table 1:** Subjects' demographics data based on their disease diagnosis.

	**Diagnosis**	**HD**	**NIND**	**OIND**	**CIS**	**RR-MS**	**P-MS**
*N*	Female/male	4/6	1/4	0/12	2/0	11/6	11/7
Age	Average	43.8	42.2	56.8	53.3	46.2	60.6
	SD	12.1	13.1	13.3	6.1	10.3	6.0
	Range	24.3–60.5	26.4–60.3	24.5–70.0	48.9–57.6	24.2–66.5	49.9–70.0
Clinical disease activity	Active/non-active	NA	NA	NA	NA	4/13	2/16
COMRIS-CTD	Average	NA	NA	NA	NA	11.7	15.9
	SD	NA	NA	NA	NA	7.4	6.2
	Range	NA	NA	NA	NA	2.2–24.2	1.5–25.1
EDSS	Average	NA	NA	NA	NA	2.4	5.3
	SD	NA	NA	NA	NA	1.6	1.8
	Range	NA	NA	NA	NA	1.0–6.5	2.5–7.5
CombiWISE	Average	NA	NA	NA	NA	20.5	43.7
	SD	NA	NA	NA	NA	12.3	16.4
	Range	NA	NA	NA	NA	6.9–51.2	20.5–70.0
MS-DSS	Average	NA	NA	NA	NA	1.3	2.2
	SD	NA	NA	NA	NA	0.8	1.0
	Range	NA	NA	NA	NA	0.5–3.4	0.5–4.0
CombiWISE slope	Average	NA	NA	NA	NA	1.7	1.9
	SD	NA	NA	NA	NA	1.5	1.5
	Range	NA	NA	NA	NA	−1.2–4.6	−1.1–4.3

### Statistical Analyses

ABs data for subjects' CSF samples were acquired with the operator blinded to subjects' clinical diagnoses. After data acquisition for all subjects, ABs per ml of CSF were compared across disease diagnostic subgroups (HD, NIND, OIND, RR-MS, and P-MS) using one-way ANOVA; as we have acquired CSF samples from only two CIS subjects, they were not included in analyses. Also, within MS subjects, ABs per ml of CSF were compared across disease activity (active vs. non-active MS) using non-parametric (Mann–Whitney) test. Within MS subjects, ABs per ml of CSF were correlated with machine-learning-optimized clinical and imaging measures of CNS tissue destruction [Composite MRI scale of CNS tissue destruction, COMRIS-CTD (31)], disability [Expanded Disability Status Scale, EDSS (32) and Combinatorial Weight-Adjusted Disability Scale, CombiWISE (33)], severity [Multiple Sclerosis Disease Severity Scale, MS-DSS (34)], and disability progression slopes (CombiWISE Slope) derived from linear regression models from CombiWISE measurements during longitudinal follow-up after LP collection using Spearman correlation analysis (GraphPad Prism 7; GraphPad Software Inc., La Jolla, CA).

## Results

### *In vitro* Model Validation

**Figure 1:**
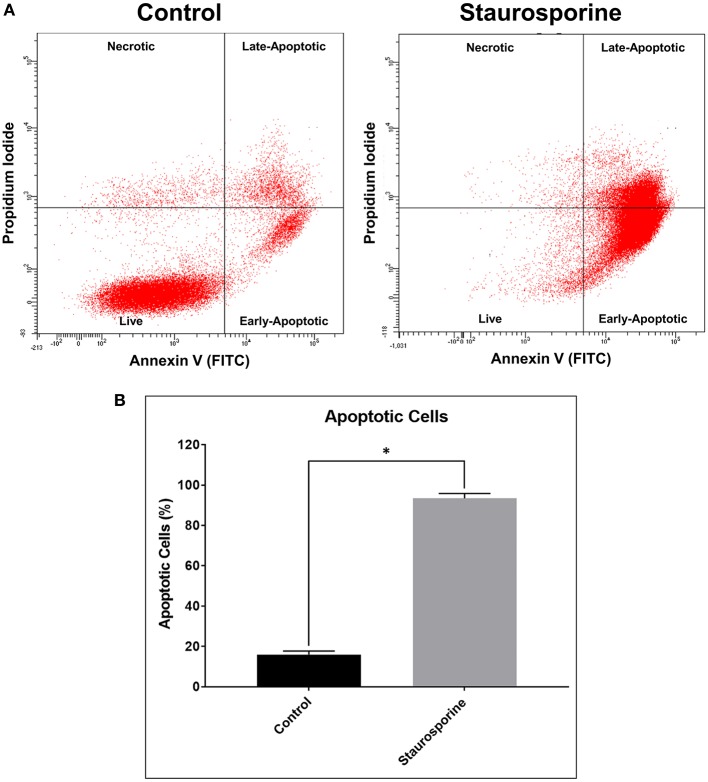
**(A)** Representative flow cytometry images of cells stained with Annexin V-FITC and propidium iodide after control or Staurosporine (0.5 μM) treatment for 24 h. **(B)** Plot of apoptotic cells (%). The error bars represent standard deviation (*n* = 6); data were analyzed using Wilcoxon test, *P* = 0.031. ^*^p < 0.05.

We validated our “identification and assessment of Abs” model using human neuronal cell line (SK-N-SH) cultures. As a positive control for induction of apoptosis we used Staurosporine treatment (0.5 μM, 24 h). Apoptotic cells were identified by staining with Annexin V and PI and were analyzed using flow cytometry. According to manufacturer's (TACS® Annexin V Kit) instructions both Annexin V and PI-negative cells are live, only Annexin V-positive cells are early-apoptotic, both Annexin V- and PI-positive cells are late-apoptotic and only PI-positive cells are necrotic (Figure 1A). After Staurosporine treatment, the % of apoptotic cells was significantly elevated (Figure 1B).

**Figure 2:**
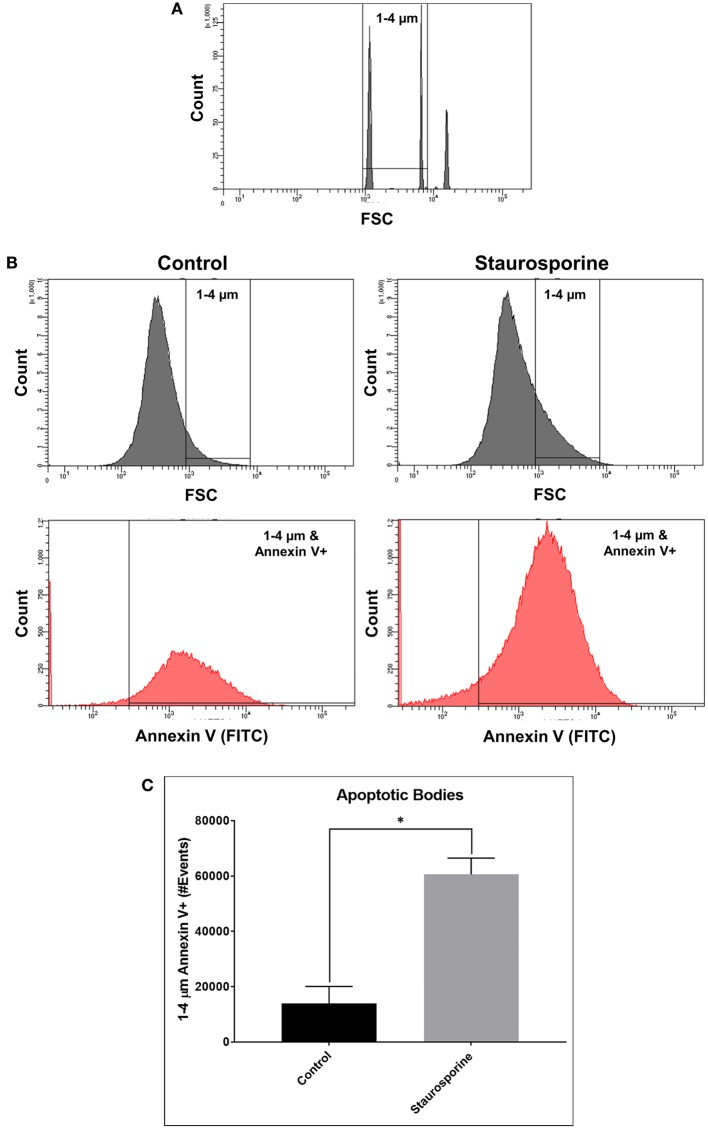
**(A)** Flow cytometry images of size calibration beads (1, 4, and 6 μm beads). **(B)** Representative flow cytometry images indicating process of ABs identification using size gate (1–4 μm) and Annexin V-FITC staining, from cell culture supernatants after control or Staurosporine (0.5 μM) treatment. **(C)** Plot of ABs (1–4 μm and Annexin V-positive events). The error bars represent standard deviation (*n* = 6); data were analyzed using Wilcoxon test, *P* = 0.031. ^*^p < 0.05.

Quantifying the induction of apoptosis by Staurosporine in our culture conditions, we next sought to quantify ABs in cell culture supernatants in order to demonstrate that our assay could differentiate between the release of ABs from control and Staurosporine-treated cultures. To this end, size gates [1–4 μm, an average size of ABs (29)] were applied in combination with Annexin V staining. First, 1–4 μm size gates were set using 1, 4, and 6 μm beads (Figure 2A; Flow Cytometry Size Calibration Kit, ThermoFisher Scientific). Within 1–4 μm vesicles ABs were identified as Annexin V positive (Figure 2B). Total ABs were quantified (1–4 μm and Annexin V-positive events); after Staurosporine treatment the total number of ABs in cell culture supernatants were significantly elevated (Figure 2C).

### Analyses of CSF Apoptotic Bodies

**Figure 3:**
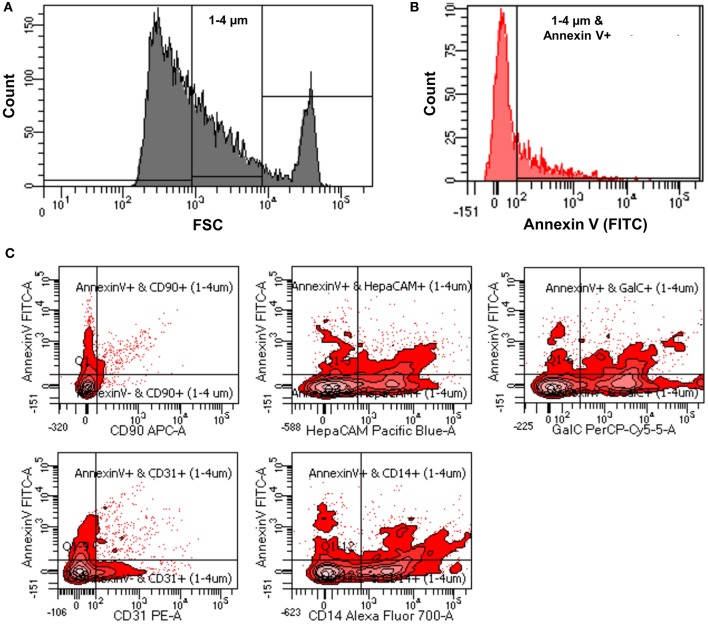
**(A)** Representative flow cytometry image indicating how size gate was used to select 1-4 μm vesicles from patients' CSF samples. **(B)** Representative flow cytometry image showing process of identification of ABs from 1 to 4 μm sized vesicles using Annexin V-FITC staining (1–4 μm and Annexin V-positive events). **(C)** Representative flow cytometry images showing how origin of ABs was identified using CNS cell-specific surface markers staining.

Verifying the flow-cytometry-based ABs detection in cell culture supernatants, we next applied the same assay to prospectively-acquired CSF samples. As described in the methods, extracellular vesicles were collected using the differential centrifugation approach. 1–4 μm vesicles were selected using size gate set by known sized beads (Figure 3A). From these vesicles total ABs were detected using Annexin V staining (1–4 μm and Annexin V-positive events; Figure 3B).

While total number of ABs in the CSF may be clinically useful, we considered the possibility that apoptosis occurs in the intrathecal compartment also under physiological conditions: e.g., any activated immune cell may cross the blood brain barrier as a part of active immunosurveillance mechanism. Such immune cell (e.g., T lymphocyte), if not re-activated in the CNS compartment, may either re-cycle back to blood via lymphatic system, or undergo apoptosis as part of physiological termination of the immune response (35, 36). Thus, we envisioned that the assay that could identify the cellular origin of ABs may have significantly higher clinical utility. To this end, we employed fluorescently-tagged antibodies specific for surface markers of different CNS cells. Selected surface markers/antibodies were previously validated as CNS cell-types-specific, because they were used to isolate specific CNS cells (i.e., neurons, oligodendrocytes, astrocytes, microglia, and endothelial cells) from human brain samples via immunopanning. Subsequent sequencing of thus-isolated CNS cell types validated that expected cell-specific transcripts were only expressed in appropriate CNS cell-type (25, 26).

The utilized cell-specific markers were: CD90+ ABs (neurons derived ABs), HepaCAM+ ABs (astrocytes derived ABs), GalC+ ABs (oligodendrocytes derived ABs), CD31+ ABs (endothelial cells derived ABs), and CD14+ ABs (myeloid cells derived ABs) (Figure 3C). Total number of ABs and cell-specific ABs were adjusted for CSF volume to obtain the number of ABs per ml of CSF (ABs/ml).

**Figure 4:**
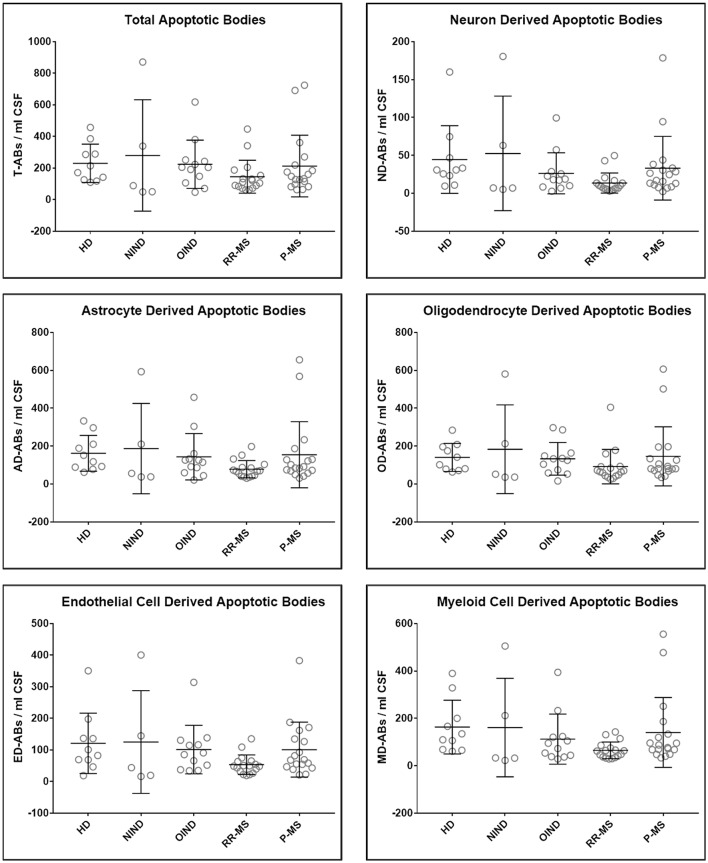
Plots of total and CNS cell-specific ABs adjusted for CSF volume (ABs/ml CSF). Each point represents individual subject; and error bars represent standard deviation (*n*: HD = 10, NIND = 5, OIND = 12, RR-MS = 17, and P-MS = 18); data were analyzed using one-way ANOVA.

**Figure 5:**
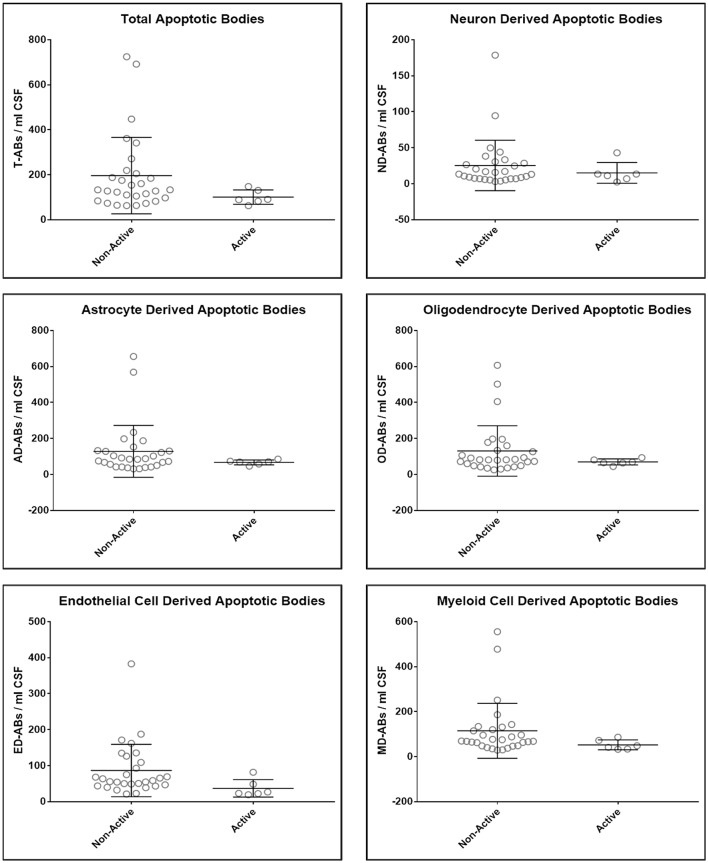
Plots of total and CNS cell-specific ABs adjusted for CSF volume (ABs/ml CSF). Each point represents individual MS subject; and error bars represent standard deviation (*n*: non-active MS = 29 and Active MS = 6); data were analyzed using nonparametric (Mann–Whitney) test.

Upon unblinding the diagnostic categories, we observed no statistically significant differences in number of total as well as CNS cell-specific ABs across disease diagnostic subgroups or MS activity (Figures 4, 5). However, while using non-overlapping cell-surface markers (i.e., each selected cell surface marker is specific for one CNS cell type and should not be expressed on any other CNS cells), we observed substantial co-expression of these markers on individual ABs. This overlap could be quantified by how much the sum of cell-specific ABs exceeds total ABs (Figure 4 and Supplementary Data File 1). Because the sum of cell-specific ABs always exceeded total number of ABs, we conclude that ABs most likely exhibited non-specific binding of antibodies. High non-specific antibody binding is a well-known problem affecting apoptotic cells, as apoptosis-induced changes in plasma cell membrane upregulate “eat me” signals recognized by phagocytes, including enhanced, non-specific binding of antibodies (37–39).

Consequently, we observed no correlations between the numbers/concentrations of total or any cell-specific ABs with accurate clinical and imaging measures CNS tissue destruction, disability, MS severity, and disability progression (Table 2).

**Table 2:** Correlation analysis (Spearman *r* and *P*-values) between adjusted total (T-ABs) and CNS cell-specific ABs (ND-ABs, neurons; AD-ABs, astrocytes; OD-ABs, oligodendrocytes; ED-ABs, endothelial cells; and MD-Abs, myeloid cells) in subjects' CSF (ABs/ml CSF) and their clinical measures of CNS tissue destruction, disability, and severity.

		**T-ABs**	**ND-ABs**	**AD-ABs**	**OD-ABs**	**ED-ABs**	**MD-ABs**
COMRIS-CTD	Spearman *r*	−0.20	−0.02	−0.10	−0.13	−0.19	−0.08
	*P*-value	0.25	0.89	0.57	0.46	0.26	0.66
EDSS	Spearman *r*	−0.07	0.23	0.07	0.01	0.07	0.15
	*P*-value	0.69	0.19	0.70	0.97	0.68	0.38
CombiWISE	Spearman *r*	−0.11	0.25	0.07	0.02	0.00	0.13
	*P*-value	0.53	0.14	0.68	0.92	0.99	0.45
MS-DSS	Spearman *r*	−0.18	0.04	−0.08	−0.09	−0.13	−0.04
	*P*-value	0.29	0.84	0.64	0.63	0.44	0.82
CombiWISE slope	Spearman *r*	−0.29	0.05	−0.05	−0.08	−0.30	−0.08
	*P*-value	0.09	0.80	0.76	0.64	0.08	0.65

## Discussion

MS has been studied extensively regarding the inflammatory component of disease (40). However, neurodegenerative component of MS, or immune-mediated destruction of specific CNS cells cannot be measured in living subjects. In this study, we attempted to analyze apoptosis in living subjects by assessing ABs in CSF. While there have been previous attempts to analyze blood/serum extracellular vesicles as markers of CNS disorders (41–43), there is no evidence for the presence of CNS ABs in blood/serum; this is likely due to their large size (0.5–4 μm) (16, 24, 29, 44) which prevents ABs from crossing the blood brain barrier (BBB) or their rapid elimination from the blood by the splenic or hepatic reticulo-endothelial system. Moreover, blood/serum naturally has a basal level of ABs from immune system cells which arise during regular immune responses (45, 46).

Our *in-vitro* studies validated that selected flow-cytometry assay measures ABs released to culture supernatants. Additionally, using CNS cell-specific surface markers previously validated in human immunopanning isolation of specific CNS cell-types provided high expectation that enumeration of CNS cell-specific ABs may be of clinical value. Unfortunately, after breaking the diagnostic codes we observed no differences between diagnostic categories and no correlations with any clinical or imaging outcomes of disability, CNS tissue destruction or MS severity. While some may argue that our study was under-powered to detect differences between diagnostic categories, we had good representation of subjects from all four diagnostic categories and found no biologically plausible trends. We concluded that expanding our dataset using the same assay would be futile, as such test could never be applied on a patient-level and therefore cannot outperform current tests such as NFL.

There are several possible interpretations of our negative results: as cell-surface proteins are often shed during apoptosis (47–49) and changes in cell membrane structure induced by apoptosis increase non-specific binding of antibodies (37–39), accurate determination of the origin of apoptotic bodies using flow cytometry may not be possible. The interference from non-specific binding is supported by the observed overlap of multiple CNS cell-type specific surface markers on the individual ABs. If non-specific antibody binding, rather than shedding of cell-surface markers from apoptotic cells was the main cause of our negative results, then attempting to use alternative reagents for detection of cell-surface molecules, such as DNA-aptamers (50) may be of use. Unfortunately, such alternative reagents are not commercially available for validated CNS cell-surface markers. Flow cytometry may also not be an ideal method for analyzing ABs, as older flow cytometers have low resolution for subcellular particles. Our employment of enhanced gating guided by size beads and validation of our assay in cell-culture supernatants mitigated this impediment.

The fate of ABs after their release from the CNS cells is unknown; while some may be secreted to the CSF via extracellular fluid, others, perhaps most, are likely phagocytosed closer to their origin (51–53). Especially, in the context of pro-inflammatory environment rich in myeloid cells such as activated microglia and infiltrating macrophages, this local capture of ABs may be much more efficient in MS and OIND controls than in healthy subject, mitigating expected differences between diagnostic categories. Thus, CSF concentrations of ABs may not reliably reflect their CNS origin.

We conclude that measuring ABs in the CSF using flow cytometry does not provide desired clinical value. We present our negative report in an effort to prevent other investigators from pursuing this path without incorporating substantial technical advancement that may mitigate problems identified in our study. Thus, a need to develop CNS cell-specific biomarkers reflective of neurodegenerative mechanisms associated with CNS diseases remains.

## Data Availability Statement

The flow cytometry data of patients' CSF ABs and their demographic and clinical information are provided in Supplementary Data File 1.

## Ethics Statement

The studies involving human participants were reviewed and approved by NIH-CNS-IRB. Written informed consent to participate in the study was provided either by participant or by his/her legal guardian/next of kin.

## Author Contributions

BB and RM designed the study. RM, JM, and JP performed the experiments. RM, PW, and BB analyzed the data. RM wrote the first draft of the manuscript. All authors contributed to manuscript revision, read, and approved the submitted version.

### Conflict of Interest

The authors declare that the research was conducted in the absence of any commercial or financial relationships that could be construed as a potential conflict of interest.

## Supplementary Material

Supplementary Data File 1 Flow cytometry data for all subjects' CSF samples including their MRI and clinical measures (COMRIS-CTD, EDSS, CombiWISE, MS-DSS, and CombiWISE Slope), clinical diagnosis and demographics (age and gender).

## References

[1] Compston A Coles A Multiple sclerosis. Lancet. (2002) 359:1221–31. 10.1016/S0140-6736(02)08220-X 11955556

[2] Friese MA Schattling B Fugger L. Mechanisms of neurodegeneration and axonal dysfunction in multiple sclerosis. Nat Rev Neurol. (2014) 10:225–38. 10.1038/nrneurol.2014.37 24638138

[3] Yilmaz A Blennow K Hagberg L Nilsson S Price RW Schouten J. Neurofilament light chain protein as a marker of neuronal injury: review of its use in HIV-1 infection and reference values for HIV-negative controls. Expert Rev Mol Diagn. (2017) 17:761–70. 10.1080/14737159.2017.1341313 28598205

[4] Khalil M Teunissen CE Otto M Piehl F Sormani MP Gattringer T. Neurofilaments as biomarkers in neurological disorders. Nat Rev Neurol. (2018) 14:577–89. 10.1038/s41582-018-0058-z 30171200

[5] Rissin DM Kan CW Campbell TG Howes SC Fournier DR Song L. Single-molecule enzyme-linked immunosorbent assay detects serum proteins at subfemtomolar concentrations. Nat Biotechnol. (2010) 28:595–9. 10.1038/nbt.1641 20495550 PMC2919230

[6] Kan CW Rivnak AJ Campbell TG Piech T Rissin DM Mosl M. Isolation and detection of single molecules on paramagnetic beads using sequential fluid flows in microfabricated polymer array assemblies. Lab Chip. (2012) 12:977–85. 10.1039/C2LC20744C 22179487

[7] Khalil M Salzer J. CSF neurofilament light: a universal risk biomarker in multiple sclerosis? Neurology. (2016) 87:1068–9. 10.1212/WNL.0000000000003107 27521432

[8] Kuhle J Barro C Disanto G Mathias A Soneson C Bonnier G. Serum neurofilament light chain in early relapsing remitting MS is increased and correlates with CSF levels and with MRI measures of disease severity. Mult Scler. (2016) 22:1550–9. 10.1177/1352458515623365 26754800

[9] Kuhle J Nourbakhsh B Grant D Morant S Barro C Yaldizli O. Serum neurofilament is associated with progression of brain atrophy and disability in early MS. Neurology. (2017) 88:826–31. 10.1212/WNL.0000000000003653 28148632 PMC5331872

[10] Bhan A Jacobsen C Myhr KM Dalen I Lode K Farbu E. Neurofilaments and 10-year follow-up in multiple sclerosis. Mult Scler. (2018) 24:1301–7. 10.1177/1352458518782005 30066611

[11] Siller N Kuhle J Muthuraman M Barro C Uphaus T Groppa S. Serum neurofilament light chain is a biomarker of acute and chronic neuronal damage in early multiple sclerosis. Mult Scler. (2019) 25:678–86. 10.1177/1352458518765666 29542376

[12] Peterson JW Bo L Mork S Chang A Trapp BD. Transected neurites, apoptotic neurons, and reduced inflammation in cortical multiple sclerosis lesions. Ann Neurol. (2001) 50:389–400. 10.1002/ana.1123 11558796

[13] Barnett MH Prineas JW. Relapsing and remitting multiple sclerosis: pathology of the newly forming lesion. Ann Neurol. (2004) 55:458–68. 10.1002/ana.20016 15048884

[14] Offen D Kaye JF Bernard O Merims D Coire CI Panet H. Mice overexpressing Bcl-2 in their neurons are resistant to myelin oligodendrocyte glycoprotein (MOG)-induced experimental autoimmune encephalomyelitis (EAE). J Mol Neurosci. (2000) 15:167–76. 10.1385/JMN:15:3:167 11303781

[15] Kerr JF Wyllie AH Currie AR. Apoptosis: a basic biological phenomenon with wide-ranging implications in tissue kinetics. Br J Cancer. (1972) 26:239–57. 10.1038/bjc.1972.33 4561027 PMC2008650

[16] Akers JC Gonda D Kim R Carter BS Chen CC. Biogenesis of extracellular vesicles (EV): exosomes, microvesicles, retrovirus-like vesicles, and apoptotic bodies. J Neurooncol. (2013) 113:1–11. 10.1007/s11060-013-1084-8 23456661 PMC5533094

[17] Mallat Z Hugel B Ohan J Leseche G Freyssinet JM Tedgui A. Shed membrane microparticles with procoagulant potential in human atherosclerotic plaques: a role for apoptosis in plaque thrombogenicity. Circulation. (1999) 99:348–53. 10.1161/01.CIR.99.3.348 9918520

[18] Werner N Wassmann S Ahlers P Kosiol S Nickenig G. Circulating CD31+/annexin V+ apoptotic microparticles correlate with coronary endothelial function in patients with coronary artery disease. Arterioscler Thromb Vasc Biol. (2006) 26:112–6. 10.1161/01.ATV.0000191634.13057.15 16239600

[19] Lazaro-Ibanez E Sanz-Garcia A Visakorpi T Escobedo-Lucea C Siljander P Ayuso-Sacido A. Different gDNA content in the subpopulations of prostate cancer extracellular vesicles: apoptotic bodies, microvesicles, and exosomes. Prostate. (2014) 74:1379–90. 10.1002/pros.22853 25111183 PMC4312964

[20] Perez-Hernandez J Cortes R. Extracellular vesicles as biomarkers of systemic lupus erythematosus. Dis Markers. (2015) 2015:613536. 10.1155/2015/613536 26435565 PMC4576008

[21] Barbour C Kosa P Komori M Tanigawa M Masvekar R Wu T. Molecular-based diagnosis of multiple sclerosis and its progressive stage. Ann Neurol. (2017) 82:795–812. 10.1002/ana.25083 29059494 PMC5743213

[22] Turiak L Misjak P Szabo TG Aradi B Paloczi K Ozohanics O. Proteomic characterization of thymocyte-derived microvesicles and apoptotic bodies in BALB/c mice. J Proteomics. (2011) 74:2025–33. 10.1016/j.jprot.2011.05.023 21635979

[23] Crescitelli R Lasser C Szabo TG Kittel A Eldh M Dianzani I. Distinct RNA profiles in subpopulations of extracellular vesicles: apoptotic bodies, microvesicles and exosomes. J Extracell Vesicles. (2013) 2:20677. 10.3402/jev.v2i0.20677 24223256 PMC3823106

[24] Szatanek R Baran J Siedlar M Baj-Krzyworzeka M. Isolation of extracellular vesicles: determining the correct approach (Review). Int J Mol Med. (2015) 36:11–7. 10.3892/ijmm.2015.2194 25902369 PMC4494580

[25] Zhang Y Chen K Sloan SA Bennett ML Scholze AR O'Keeffe S. An RNA-sequencing transcriptome and splicing database of glia, neurons, and vascular cells of the cerebral cortex. J Neurosci. (2014) 34:11929–47. 10.1523/JNEUROSCI.1860-14.2014 25186741 PMC4152602

[26] Zhang Y Sloan SA Clarke LE Caneda C Plaza CA Blumenthal PD. Purification and characterization of progenitor and mature human astrocytes reveals transcriptional and functional differences with mouse. Neuron. (2016) 89:37–53. 10.1016/j.neuron.2015.11.013 26687838 PMC4707064

[27] Liu L Shi GP. CD31: beyond a marker for endothelial cells. Cardiovasc Res. (2012) 94:3–5. 10.1093/cvr/cvs108 22379038

[28] Ziegler-Heitbrock HW Ulevitch RJ. CD14: cell surface receptor and differentiation marker. Immunol Today. (1993) 14:121–5. 10.1016/0167-5699(93)90212-4 7682078

[29] Hristov M Erl W Linder S Weber PC. Apoptotic bodies from endothelial cells enhance the number and initiate the differentiation of human endothelial progenitor cells *in vitro*. Blood. (2004) 104:2761–6. 10.1182/blood-2003-10-3614 15242875

[30] Polman CH Reingold SC Banwell B Clanet M Cohen JA Filippi M. Diagnostic criteria for multiple sclerosis: 2010 revisions to the McDonald criteria. Ann Neurol. (2011) 69:292–302. 10.1002/ana.22366 21387374 PMC3084507

[31] Kosa P Komori M Waters R Wu T Cortese I Ohayon J. Novel composite MRI scale correlates highly with disability in multiple sclerosis patients. Mult Scler Relat Disord. (2015) 4:526–35. 10.1016/j.msard.2015.08.009 26590659 PMC4656129

[32] Kurtzke JF. Rating neurologic impairment in multiple sclerosis: an expanded disability status scale (EDSS). Neurology. (1983) 33:1444–52. 10.1212/WNL.33.11.1444 6685237

[33] Kosa P Ghazali D Tanigawa M Barbour C Cortese I Kelley W. Development of a sensitive outcome for economical drug screening for progressive multiple sclerosis treatment. Front Neurol. (2016) 7:131. 10.3389/fneur.2016.00131 27574516 PMC4983704

[34] Weideman AM Barbour C Tapia-Maltos MA Tran T Jackson K Kosa P. New multiple sclerosis disease severity scale predicts future accumulation of disability. Front Neurol. (2017) 8:598. 10.3389/fneur.2017.00598 29176958 PMC5686060

[35] Weller RO Engelhardt B Phillips MJ. Lymphocyte targeting of the central nervous system: a review of afferent and efferent CNS-immune pathways. Brain Pathol. (1996) 6:275–88. 10.1111/j.1750-3639.1996.tb00855.x 8864284

[36] Engelhardt B Carare RO Bechmann I Flugel A Laman JD Weller RO. Vascular, glial, and lymphatic immune gateways of the central nervous system. Acta Neuropathol. (2016) 132:317–38. 10.1007/s00401-016-1606-5 27522506 PMC4992028

[37] Bailey RW Nguyen T Robertson L Gibbons E Nelson J Christensen RE. Sequence of physical changes to the cell membrane during glucocorticoid-induced apoptosis in S49 lymphoma cells. Biophys J. (2009) 96:2709–18. 10.1016/j.bpj.2008.12.3925 19348753 PMC2711280

[38] Gibbons E Pickett KR Streeter MC Warcup AO Nelson J Judd AM. Molecular details of membrane fluidity changes during apoptosis and relationship to phospholipase A(2) activity. Biochim Biophys Acta. (2013) 1828:887–95. 10.1016/j.bbamem.2012.08.024 22967861 PMC3529823

[39] Zhang Y Chen X Gueydan C Han J. Plasma membrane changes during programmed cell deaths. Cell Res. (2018) 28:9–21. 10.1038/cr.2017.133 29076500 PMC5752838

[40] Buck D Hemmer B. Treatment of multiple sclerosis: current concepts and future perspectives. J Neurol. (2011) 258:1747–62. 10.1007/s00415-011-6101-2 21637950

[41] Garcia-Romero N Carrion-Navarro J Esteban-Rubio S Lazaro-Ibanez E Peris-Celda M Alonso MM. DNA sequences within glioma-derived extracellular vesicles can cross the intact blood-brain barrier and be detected in peripheral blood of patients. Oncotarget. (2017) 8:1416–28. 10.18632/oncotarget.13635 27902458 PMC5352065

[42] Selmaj I Cichalewska M Namiecinska M Galazka G Horzelski W Selmaj KW. Global exosome transcriptome profiling reveals biomarkers for multiple sclerosis. Ann Neurol. (2017) 81:703–17. 10.1002/ana.24931 28411393

[43] Galazka G Mycko MP Selmaj I Raine CS Selmaj KW. Multiple sclerosis: serum-derived exosomes express myelin proteins. Mult Scler. (2018) 24:449–58. 10.1177/1352458517696597 28273783

[44] Ihara T Yamamoto T Sugamata M Okumura H Ueno Y. The process of ultrastructural changes from nuclei to apoptotic body. Virchows Arch. (1998) 433:443–7. 10.1007/s004280050272 9849859

[45] Caruso S Poon IKH. Apoptotic cell-derived extracellular vesicles: more than just debris. Front Immunol. (2018) 9:1486. 10.3389/fimmu.2018.01486 30002658 PMC6031707

[46] Xu X Lai Y Hua ZC. Apoptosis and apoptotic body: disease message and therapeutic target potentials. Biosci Rep. (2019) 39:BSR20180992. 10.1042/BSR20180992 30530866 PMC6340950

[47] Madge LA Sierra-Honigmann MR Pober JS. Apoptosis-inducing agents cause rapid shedding of tumor necrosis factor receptor 1 (TNFR1). A nonpharmacological explanation for inhibition of TNF-mediated activation. J Biol Chem. (1999) 274:13643–9. 10.1074/jbc.274.19.13643 10224136

[48] Ilan N Mohsenin A Cheung L Madri JA. PECAM-1 shedding during apoptosis generates a membrane-anchored truncated molecule with unique signaling characteristics. FASEB J. (2001) 15:362–72. 10.1096/fj.00-0372com 11156952

[49] DeLeo FR. Attractive shedding. Blood. (2007) 110:1711–2. 10.1182/blood-2007-06-096677 22993882 PMC1976359

[50] Giudice V Biancotto A Wu Z Cheung F Candia J Fantoni G. Aptamer-based proteomics of serum and plasma in acquired aplastic anemia. Exp Hematol. (2018) 68:38–50. 10.1016/j.exphem.2018.09.008 30312735 PMC6748047

[51] Witting A Muller P Herrmann A Kettenmann H Nolte C. Phagocytic clearance of apoptotic neurons by Microglia/Brain macrophages *in vitro*: involvement of lectin-, integrin-, and phosphatidylserine-mediated recognition. J Neurochem. (2000) 75:1060–70. 10.1046/j.1471-4159.2000.0751060.x 10936187

[52] Takahashi K Rochford CD Neumann H. Clearance of apoptotic neurons without inflammation by microglial triggering receptor expressed on myeloid cells-2. J Exp Med. (2005) 201:647–57. 10.1084/jem.20041611 15728241 PMC2213053

[53] Kurant E Axelrod S Leaman D Gaul U. Six-microns-under acts upstream of Draper in the glial phagocytosis of apoptotic neurons. Cell. (2008) 133:498–509. 10.1016/j.cell.2008.02.052 18455990 PMC2730188

